# Biocompatible Magnetopyroelectric Composite Films for Cell Stimulation

**DOI:** 10.1002/advs.202520491

**Published:** 2026-02-04

**Authors:** Hao Ye, Joaquin Llacer‐Wintle, Semih Sevim, Elric Zhang, Denis von Arx, Lukas Hertle, Martina Accursi, Minsoo Kim, Josep Puigmartí‐Luis, Bradley J. Nelson, Xiang‐Zhong Chen, Salvador Pané

**Affiliations:** ^1^ Multi‐Scale Robotics Lab (MSRL) Institute of Robotics & Intelligent Systems (IRIS) Zurich Switzerland; ^2^ Departament De Ciència dels Materials i Química Física Institut De Química Teòrica i Computacional University of Barcelona Barcelona Spain; ^3^ Institució Catalana de Recerca i Estudis Avançats (ICREA) Pg. Lluís Companys 23 Barcelona Spain; ^4^ International Institute for Intelligent Nanorobots and Nanosystems College of Intelligent Robotics and Advanced Manufacturing State Key Laboratory of Photovoltaic Science and Technology Shanghai Frontiers Science Research Base of Intelligent Optoelectronics and Perception and Institute of Optoelectronics Fudan University Shanghai China; ^5^ Zhejiang Key Laboratory of Extreme Environment Functional Materials Yiwu Research Institute of Fudan University Yiwu China

**Keywords:** biocompatible magnetoelectric materials, Iron oxide nanoparticles, Magnetopyroelectricity, neural progenitor cell differentiation, PVDF‐TrFE composite films

## Abstract

Magnetoelectric materials, which generate electric fields in response to alternating magnetic stimulation, are increasingly recognized for their applications in neuromodulation, tissue engineering, wireless drug delivery, and cancer treatment. This study addresses the cytotoxicity concerns associated with heavy metals in traditional magnetoelectric composites by introducing a heat‐mediated magnetoelectric approach utilizing biocompatible iron oxide nanoparticles and pyroelectric polymers, thereby enhancing biomedical safety. The nanoparticles were synthesized with controlled size and shape via thermal decomposition of iron oleate, employing an in situ temperature labeling technique that simplifies the synthesis process and ensures uniform particle formation. These nanoparticles, optimized for high heating efficiency, were combined with the pyroelectric polymer P(VDF‐TrFE) to create composite films that exhibit a heat‐mediated magnetoelectric effect. This effect involves an alternating magnetic field heating the nanoparticles, leading to reversible material depolarization and the generation of a pyroelectric current. We explored the magnetopyroelectric effect on cell differentiation, demonstrating excellent biocompatibility with neural progenitor cells and significant enhancement in neuronal differentiation, attributed to the synergistic effects of heat and electricity. The pro‐differentiation mechanism of magnetopyroelectric stimulation involves phosphatidylinositol 3 kinase AKT pathway and calcium signaling. This heat‐mediated magnetoelectric approach not only presents a potential for applications such as neuronal repair and targeted drug delivery but also provides a safer and more versatile alternative to conventional magnetoelectric materials.

## Graphical abstract

Magnetopyroelectric films couple Fe3O4 nanoparticles with pyroelectric P(VDF‐TrFE) to turn alternating magnetic fields into controlled heat and pyroelectric currents. This heavy‐metal free platform safely boosts neural progenitor cells differentiation via synergistic thermal and pyroelectrical cues, engages AKT and calcium pathways, and opens practical routes for neuromodulation and targeted delivery.

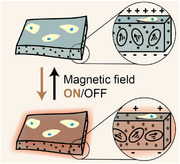

## 1 Introduction

Electrical signals are intrinsic to cellular function, orchestrating activities from communication to complex physiological responses across various biological systems [1]. Recognizing the potential of these signals, recent therapeutic strategies have focused on replicating endogenous electrical cues to foster an environment akin to healthy tissue, aiming to restore or enhance impaired cellular functions [2, 3]. Complementarily, magnetic stimulation can modulate ion‐channel activity and mechanotransduction to influence cell behavior [4]. Such applications include pacemakers to compensate for inadequate natural heart pulses [5], external electrical stimulation to guide cell migration in wound healing [6], and deep brain stimulation (DBS) for managing neural activity [7, 8, 9]. Despite the promising prospects of electrical stimulation, the precise delivery of these signals within the central nervous system presents substantial challenges, requiring sophisticated technologies for accurate control [10, 11].

Magnetoelectric materials, designed to transduce magnetic stimuli into electrical signals, offer a pathway for non‐invasive therapeutic applications [12, 13]. Previous reports have demonstrated that the electric fields caused by actuated magnetoelectric composites can promote proliferation and differentiation of neuroblasts [12, 14, 15, 16, 17], osteoblasts [18, 19, 20], and myoblasts [21, 22]. Moreover, by activating magnetoelectric materials with an external magnetic field, it is possible to achieve a targeted and timed release of drugs, reducing side effects and improving the efficacy of treatments [23, 24, 25, 26]. Across noninvasive transduction strategies that include magnetoelectric composites, photothermal–pyroelectric systems, and ultrasound driven piezoelectrics, practical deployment is subject to modality‐specific physical limits. Magnetoelectric composites depend on carefully engineered interfaces to transfer strain, a requirement that becomes especially challenging when manufacturing nanometer‐scale films, nanocomposites, and core–shell particles. Their operation is further constrained by mechanical resonances and interfacial losses, which narrow the usable frequency range [27, 28]. Photothermal–pyroelectric strategies, in turn, require optical access, and therefore suffer from limited penetration in confined tissues [29]. Ultrasound‐driven piezoelectric stimulation avoids the optical constraint but is attenuated by several tissues (e.g., bone) and can, in some cases, require invasive access to deliver sufficient energy [30, 31, 32]. In addition to these physics constraints, ensuring that the deployment of these materials is safe within biological systems is of foremost importance, yet all currently available examples of magnetoelectric composites rely on the structural incorporation of heavy metal ions such as bismuth, lead, cobalt, and nickel, whose degradation results in the release of highly cytotoxic and potentially carcinogenic ions [33].

**FIGURE 1:**
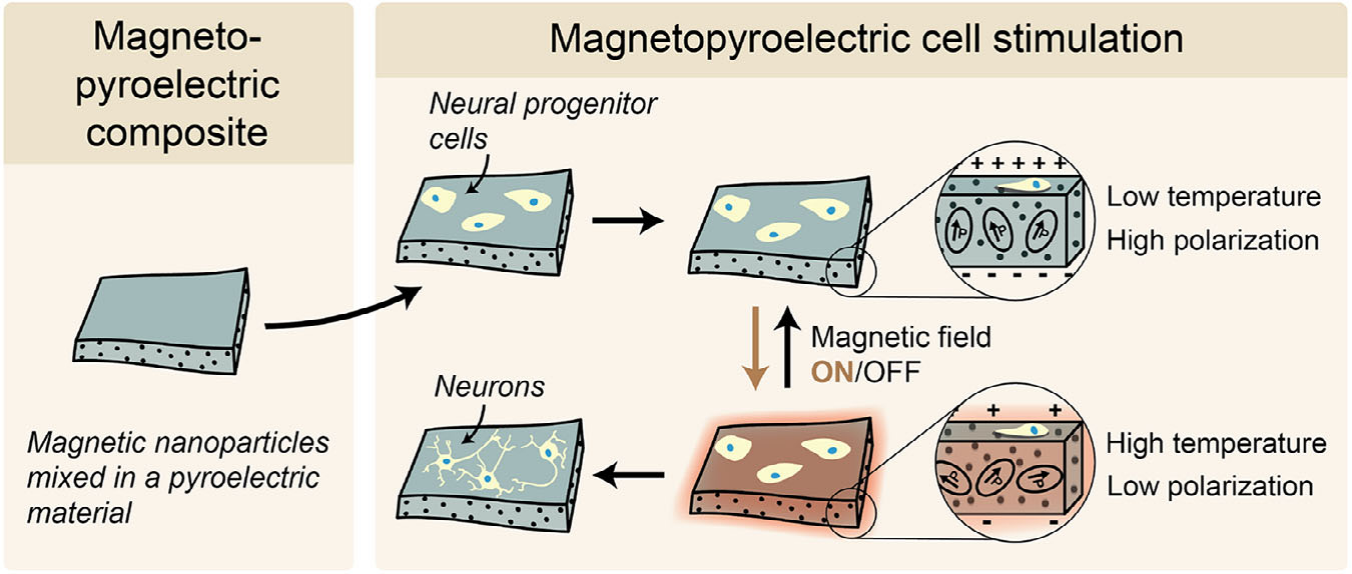
Schematic representation of magnetopyroelectric films for cell stimulation. The magnetopyroelectric effect results from the synergistic interaction between magnetic particle heating and pyroelectricity. In this process, an alternating magnetic field induces magnetic hysteresis in magnetic nanoparticles, leading to their heating. This thermal effect causes reversible depolarization of the pyroelectric material. Repeated exposure to magnetopyroelectric‐induced changes in temperature (37°C – 41°C) and polarization could be used to induce the differentiation of neural progenitor cells.

To address both the physical and biocompatibility limitations, our study exploits a heat‐mediated magnetoelectric approach that exploits composites comprising magnetothermal iron oxide nanoparticles (IONPs) embedded in a pyroelectric polymer P(VDF‐TrFE) matrix [34]. The synthesis of IONPs through the thermal decomposition of iron oleate is optimized to achieve precise control over particle size and shape, which is crucial for the efficiency of the heat‐mediated magnetoelectric effect. When an alternating magnetic field (AMF) is applied to these composites, the induced magnetic hysteretic losses heat the nanoparticles, which in turn transiently depolarize the pyroelectric matrix and generate a pyroelectric current. This study demonstrates the application of these magnetopyroelectric materials in a biomedical context, particularly focusing on the differentiation of neural progenitor cells (NPCs, Figure 1). By employing a designed stimulation protocol, we found that the heat and electricity generated by magnetopyroelectricity induces neural progenitor cell differentiation in a synergistic fashion that is greater than that induced by either heat or electricity alone, as evidenced by the expression of neural markers βIII‐tubulin and microtubule‐associated protein 2 (MAP2). Furthermore, our investigation explores the molecular mechanisms underpinning this enhanced differentiation and provides evidence for the involvement of the phosphatidylinositol 3‐kinase (PI3K)‐AKT pathway and calcium signaling. Specifically, we found that both pathways are required and act cooperatively under AMF stimulation, as their simultaneous inhibition caused a synergistic suppression of neuronal differentiation, while pharmacological rescue confirmed their functional relevance. The results underscore the synergy of heat and electric stimulation, offering a promising method to guide NPCs differentiation toward specific neural lineages. The compelling biocompatibility characteristics of these pyroelectric materials, evidenced by NPC viability exceeding 90%, highlights their potential for applications in neural regeneration and repair. By introducing safer magnetoelectric materials, we broaden the therapeutic possibilities in neuromodulation, tissue engineering, and targeted drug delivery, and may also facilitate a faster transition to clinical applications.

## 2 Results and Discussion

### 2.1 Characterization of MPE Films

**FIGURE 2:**
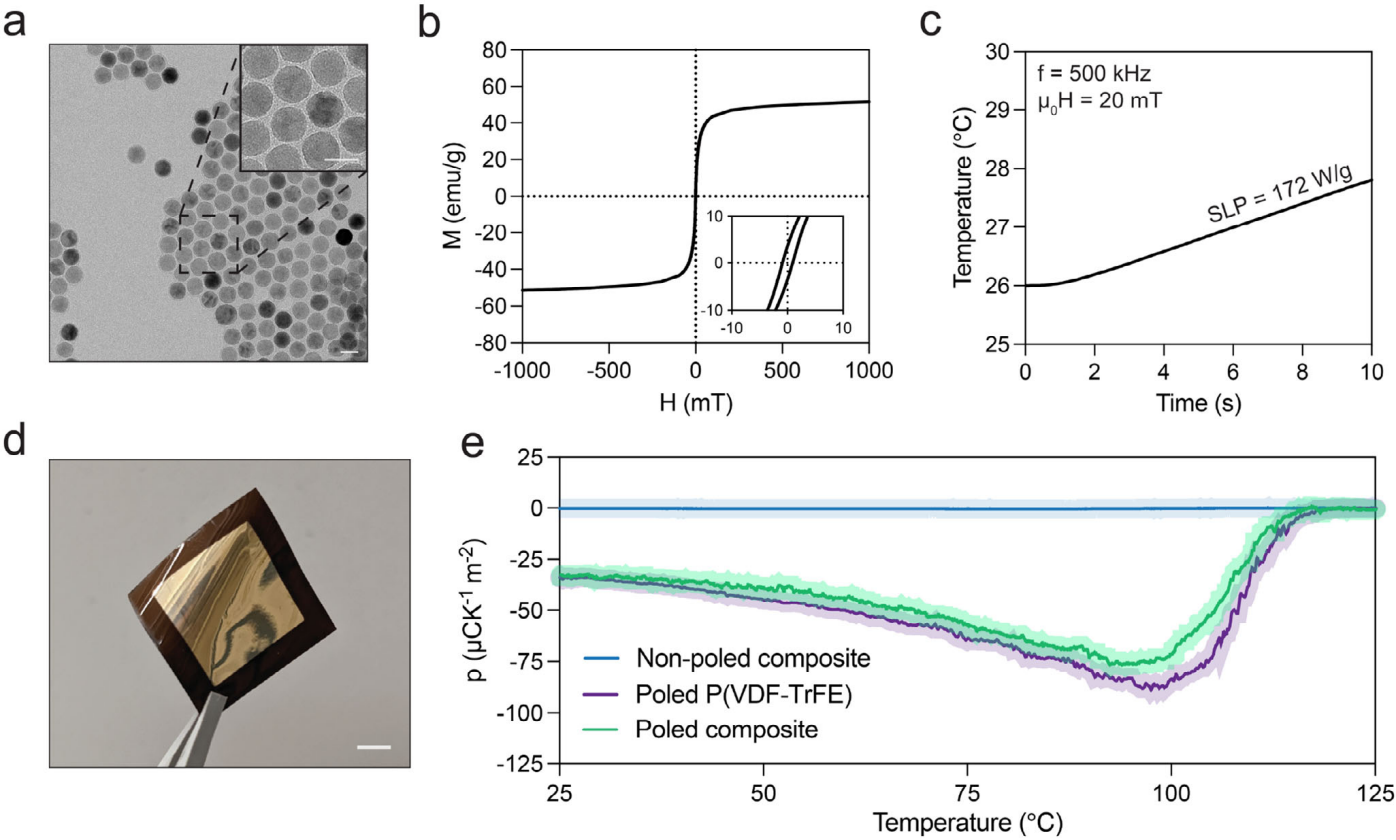
Characterization of the magnetopyroelectric films used for cell stimulation. (a) Transmission electron microscopy (TEM) image of the IONPs, scale bar = 25 nm. (b) Magnetic hysteresis loop of the IONPs. The inset shows the curve from ‐10 mT to 10 mT. (c) Magnetic heating of the iron oxide nanoparticles: the time‐dependent temperature variation under an alternating magnetic field. (d) A typical magnetopyroelectric composite film with gold‐evaporated electrodes used for characterization. Scale bar = 10 mm. (e) Pyroelectric coefficient as a function of the temperature of films with and without IONPs and with different poling treatments, obtained via the Sharp‐Garn method (n = 3).

The composite films incorporate magnetic IONPs, produced through the thermal decomposition of iron oleate in eicosane [34, 35] (Figure S1). Transmission electron microscopy (TEM) depict these nanoparticles as spherical (Figure 2a) with a uniform size distribution (Polydispersity Index, PDI = 0.12) and an average diameter of 21.4 ± 1.1 nm. Vibrating sample magnetometry (VSM) (Figure 2b) shows that IONPs exhibit a coercive field (µ_0_H_C_) of 0.9 mT and a saturation magnetization (M_S_) of 52 emu g^−1^ As is typical for particles produced via thermal decomposition, their defective crystal structure leads to reduced magnetization compared with bulk magnetite and maghemite [36, 37]. The specific loss power (SLP) of IONPs, indicative of their heating efficiency, was quantified by the temperature rise observed in a water‐based IONPs dispersion under an AMF (20 mT, 500 kHz). Figure 2c shows that an SLP of 172 W g^−1^ is equivalent to a temperature increase of approximately 2°C in 10 s.

Composite films comprising a 10 wt.% of IONPs in P(VDF‐TrFE) were prepared using a doctor blade technique (Figure S2). After post‐annealing and poling steps, these films exhibited an average thickness of 53 ± 5 µm, and displayed homogeneity, flexibility, and a brown coloration (Figure 2d; Figure S3). The indirect Sharp‐Garn approach was used to determine the pyroelectric coefficient by measuring the correlation between temperature and spontaneous polarization variations. This technique involves monitoring the current produced by a sample as its temperature is varied sinusoidally, according to

(1) $$T\;\left(t\right)=T_{amp}\;\sin\left(\omega t\right)+at+b$$

where *T_amp_* represents the amplitude of temperature oscillation, *t* denotes time, *ω* is the angular frequency of oscillation, *a* indicates the slope, and *b* corresponds to the offset [38]. The calculation for the pyroelectric coefficient is

(2) $$p\;\left(T\right)=\frac{I_{AMP}\times \sin\emptyset }{A\times \omega \times T_{AMP}}\;$$

where *I_AMP_* and ∅ are the current amplitude and phase offset, respectively. This methodology precisely characterizes the pyroelectric properties over a temperature range and mitigates non‐pyroelectric effects such as strain‐induced piezoelectric and flexoelectric currents [38, 39]. The pyroelectric coefficients of poled and unpoled films were determined from current measurements performed between 25°C and 125°C, revealing no pyroelectric activity in non‐poled films across the temperature spectrum and negligible pyroelectric coefficients (Figure 2e). In contrast, poled films displayed pyroelectric coefficients of approximately ‐30 µC K^−1^ m^−2^ at 25°C, decreasing to about ‐90 µC K^−1^ m^−2^ at 100°C. The films lost their spontaneous polarization at temperatures above this, and the pyroelectric coefficient approached zero as the P(VDF‐TrFE) underwent a phase transition from ferroelectric to paraelectric state [40]. At 125°C, the films displayed 0 µC K^−1^ m^−2^.

### 2.2 Characterization of MPE Coupling in Films Used for Cell Stimulation

To demonstrate the magnetopyroelectric coupling in films comprised of P(VDF‐TrFE) and IONPs, we next monitored the temperature fluctuations triggered by a pulsed AMF and correlated these to the pyroelectric current measured in a closed circuit. The experimental apparatus included a magnetic coil for field generation, a thermal camera to observe film temperature, and an electrometer for current measurement, with the films positioned centrally within the coil to minimize eddy currents and the electrodes being parallel to the direction of the field (Figure S4). The films, upon exposure to a pulsed AMF, are expected to undergo heating via the IONPs' hysteretic losses and cooling through air convection, reaching thermal equilibrium and generating an electrical current proportionate to the rate of temperature changes due to their pyroelectric properties [34].

**FIGURE 3:**
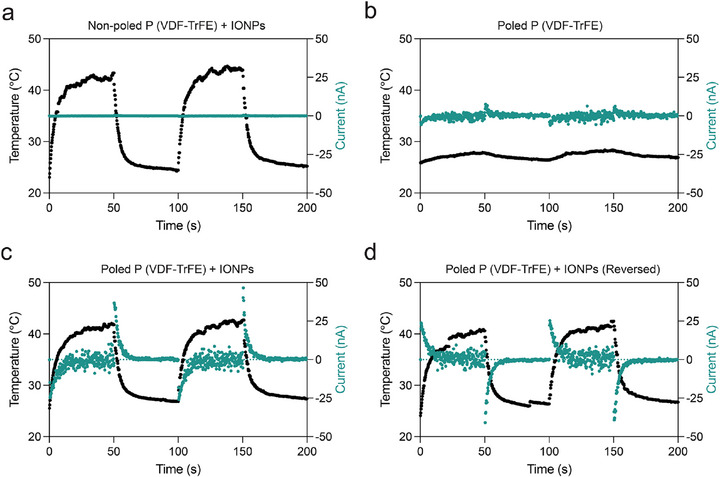
Demonstration of magnetopyroelectric coupling in films utilized for cell stimulation. Upon application of a pulsed alternating magnetic field (µ_0_H = 20 mT, f = 500 kHz, T_pulse_ = 100 s), both temperature and current were measured for (a) a non‐poled P(VDF‐TrFE) film with IONPs, (b) a poled P(VDF‐TrFE) film, and (c) a poled P(VDF‐TrFE) film with IONPs in forward orientation, as well as (d) in reversed orientation.

The heat‐mediated magnetoelectricity was assessed by measuring the electrical current and temperature across a composite film subjected to a pulsed AMF, as depicted in Figure 3. Specifically, an initial experiment involved a non‐poled film with 10 wt.% IONPs, which acted as a negative control group. This setup exhibited a rapid temperature increase, achieving a maximum rise (ΔT) of 22°C within 50 s of exposure to the magnetic field (Figure 3a), yet no pyroelectric current was observed. Additionally, in a negative control group where a poled film without IONPs was subjected to identical conditions, there was a modest temperature increase of ∼3°C, attributed primarily to induction heating in the electrodes. This scenario produced a low current, indicating that the poled P(VDF‐TrFE) films have a slight pyroelectric effect (Figure 3b). Similarly, experiments with a P(VDF‐TrFE) non‐poled film also without IONPs displayed a comparable modest temperature increase of ∼4°C, but with no detectable pyroelectric current (Figure S5). Further experiments involved a poled composite film (10 wt.% IONPs) under the same experimental conditions. The incorporation of IONPs significantly enhanced the rate of temperature change, which, when coupled with the pyroelectric properties of the P(VDF‐TrFE) poled films, resulted in peak currents of around ‐50 and ‐25 nA while cooling and heating respectively, compared to the control groups (Figure 3c). These findings point to the existence of magnetopyroelectric coupling in the composite films, in which the IONPs are magnetically heated by the AMF to produce a pyroelectric current. By taking measurements in both standard and flipped film orientations, we were able to confirm that the electrical signals were from the film (Figure 3c,d). The magnetopyroelectric origin of the signal was confirmed by a corresponding switch in current direction that occurred when the film orientation was reversed. Using a specified equation, the film's pyroelectric coefficient (T_pulse_ = 100 s) was calculated from the magnetopyroelectric response [34]

(3) $$p\;\left(T\right)=\frac{I_{p}}{A\times dT/dt}\;$$

The pyroelectric current, electrode area, and rate of temperature change are denoted by *I_p_*, *A*, and *dT*/*dt*, respectively. The latter was derived by fitting the temperature curves during heating and cooling to a two‐phase exponential model and computing the respective derivatives. The derived pyroelectric coefficients closely matched those predicted by the Sharp‐Garn approach, corroborating the pyroelectric characteristics of the measured currents (Figure S6, the corresponding fits are shown in Figure S7a–c). As depicted in Figure S7d, the integration of magnetic and pyroelectric effects in the magnetopyroelectric films facilitates the precise manipulation of thermal and electrical fields. This control is achieved by adjusting the parameters of the magnetic field, thereby realizing the targeted applications in biomedicine. In addition, to isolate the thermal component of AMF stimulation, we prepared non‐poled P(VDF‐HFP) + IONPs composite films deliberately stabilized in the non‐polar α phase. X‐ray diffraction confirmed the α‐type signature and the absence of characteristic β‐phase reflections (Figure S8), indicating negligible pyroelectric contributions. Under AMF, we used this material to implement strictly thermal stimulation control.

### 2.3 Biocompatibility Assessment of MPE Films

**FIGURE 4:**
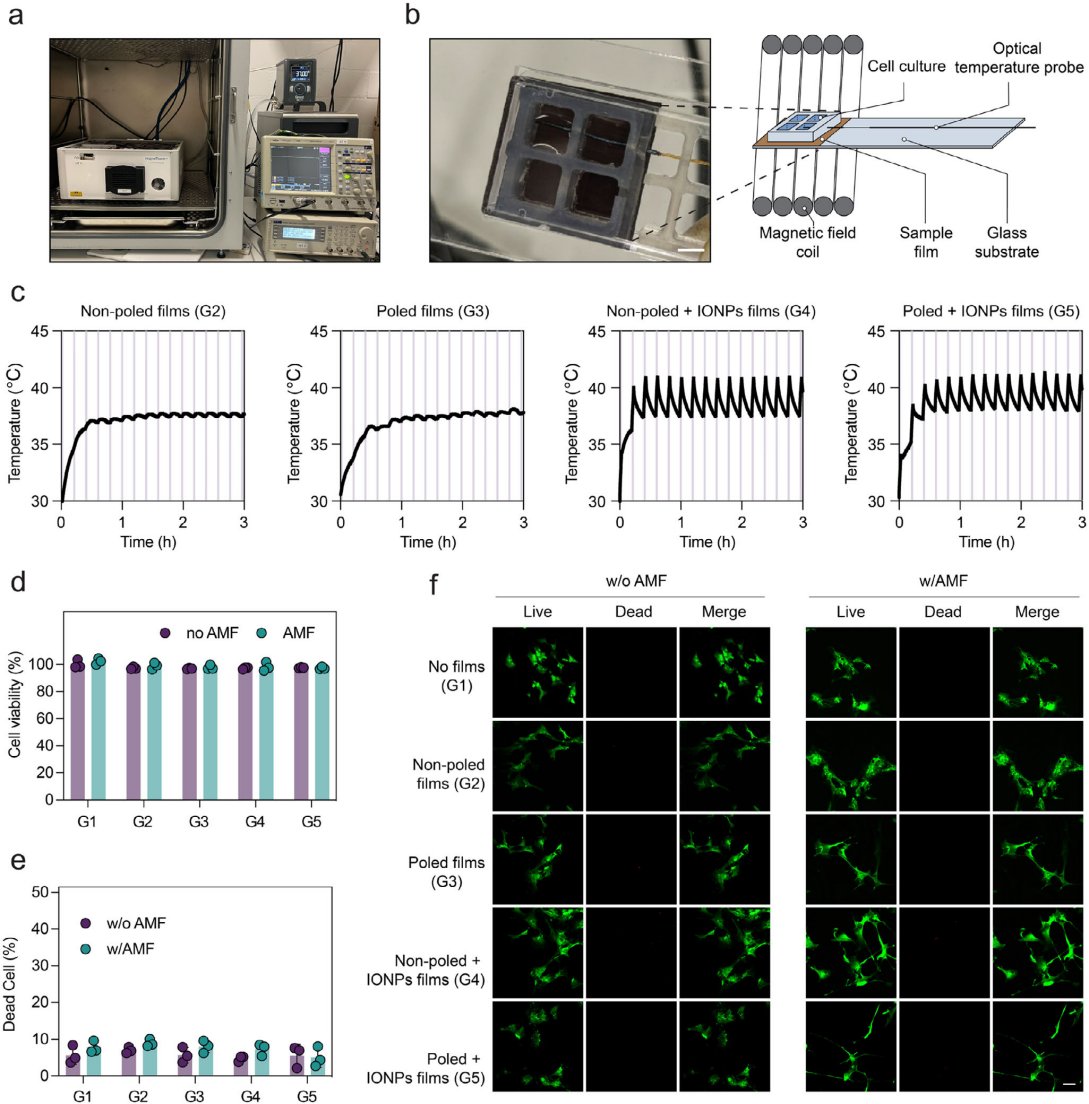
Biocompatibility of magnetopyroelectric films. (a) The whole setup is mounted inside an incubator. (b) The sample (brown) is placed between a glass slide and a silicon multiwell separator. The optical thermometer was submerged in cell medium and in close contact with the sample film (Scale bar = 7 mm). (c) Representative temperature profiles recorded by the optical thermometer during MPE cell stimulation for different samples. (d) MTT assay evaluating the effect of MPE films on the viability of NPCs subjected to an AMF applied twice daily for two days (n = 3). (e) NPC viability assessed through a live/dead assay under various conditions, with quantification (n = 3) and confocal imaging (f) of the assay results. The scale bar is 50 µm.

Motivated by the magnetopyroelectric properties observed, we hypothesize that poled P(VDF‐TrFE) films embedded with IONPs may serve as magnetopyroelectric platforms for neural repair. Individual modalities based on thermal and electrical stimulation have shown efficacy in promoting the differentiation of NPCs and stem cells [41, 42]. However, the synergistic effects of simultaneous thermal and electrical stimulation on cellular differentiation remain unexplored. To investigate this, we have developed a system designed for investigating cellular magnetopyroelectric stimulation, depicted in Figure 4a. This system comprised 26 × 26 mm square composite films strategically positioned between a glass slide and a multiwell silicon chamber. Within this configuration, the films were segmented into four quadrants: three served as experimental replicates, and one facilitated temperature monitoring using an optical sensor (Figure 4b). The setup was housed in a controlled incubator environment where a periodic application of an AMF was executed (T_pulse_ = 708 s, AMF duty cycle = 16.1%, frequency = 500 kHz, µ_0_H = 20 mT). This specific pulse regime and duty cycle were optimized to maintain the temperature of the cell medium within a narrow range of 37°C to 41°C, which is a critical measure to circumvent cellular mortality [42]. Temperature profiles for each sample type were recorded, showcasing control over temperature fluctuations (Figure 4c). The cell stimulation protocol was carried out over two consecutive days, each encompassing two 3‐h stimulation sessions (i.e., one in the morning and one in the afternoon).

As shown in Figure S9, the NPC stemness using Nestin and Sox2 markers with 98.8% and 99.0% positive fractions via flow cytometry analysis, respectively. Cell viability, utilizing NPCs, was evaluated through the MTT (3‐(4, 5‐dimethylthiazol‐2‐yl)‐2,5‐diphenyltetrazolium bromide) assay to explore the effects of film composition, poling orientation, and AMF application after 48 h of incubation, as shown in Figure 4d–f. The viability of the control groups, which did not include films (G1), established a baseline that remained stable in the presence of AMF. This consistency was observed across various configurations, including poled and non‐poled P(VDF‐TrFE) films, with or without IONPs, maintaining cell viability above 90%. These results indicate a lack of adverse effects on cellular health. Fluorescence imaging from the Live/Dead assay further corroborated these outcomes by demonstrating preserved cellular membrane integrity under all experimental conditions, with less than 10% cell mortality noted, thereby affirming the high biocompatibility of the magnetopyroelectric composites (Figure 4e,f). Notably, confocal fluorescence imaging also revealed obvious morphological changes in cells interfaced with poled films (G3) and both poled and non‐poled composite films (G4 & G5) under AMF exposure, suggesting possible differentiation of NPCs triggered by the combined thermal and magnetopyroelectric stimuli. We next evaluated long‐term cytocompatibility across six experimental groups and various stimulation regimes by MTT assay. Cell viability remained high in every condition, consistently above 90%, indicating that both the thermal and magnetopyroelectric stimulation are well tolerated by NPCs over the tested timeframes (Figure S10).

### 2.4 Cell Stimulation With MPE Films

**FIGURE 5:**
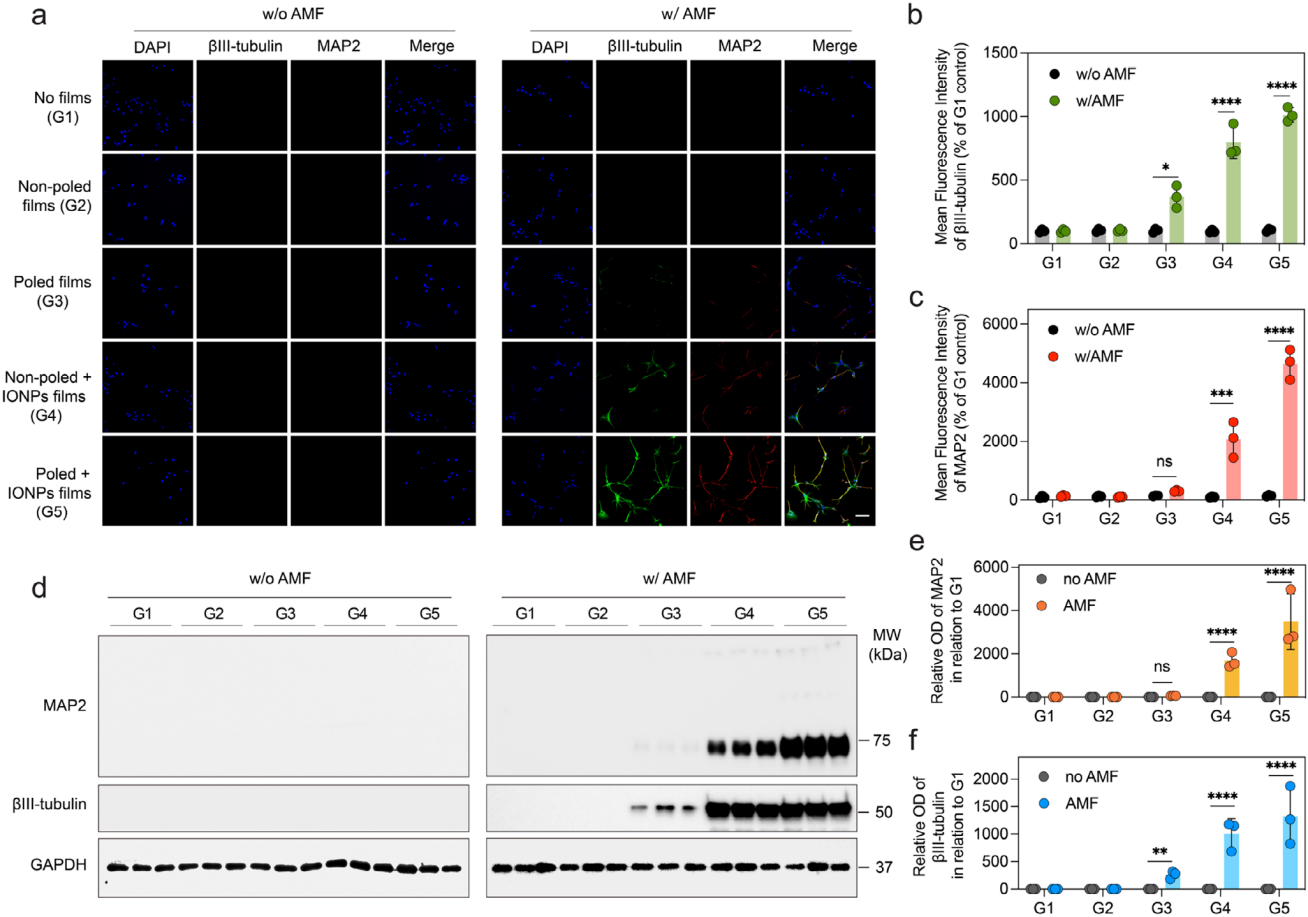
Cell stimulation with MPE films. (a) Immunofluorescence assay evaluating the effect of MPE films on NPC differentiation. DAPI (blue), βIII‐tubulin (green), and MAP2 (red) serve as markers to monitor the progression of NPCs into neurons. The scale bar is set at 50 µm. Statistical analyses of protein expression levels for (b) βIII‐tubulin and (c) MAP2 are provided (n = 3). (d) Western blot evaluating the effect of MPE films on NPC differentiation. Quantification of (e) βIII‐tubulin and (f) MAP2 expression under AMF post‐treatment of each samples are provided (n = 3). Statistical significance was calculated via one‐way ANOVA with a Tukey post‐hoc test (Figure 5b, c, e, f).^*^ *p* < 0.05, ^**^ *p* < 0.01, ^***^ *p* < 0.001, ^****^ *p* < 0.0001 versus control.

The effect of magnetopyroelectric stimulation on cell differentiation was assessed by measuring the cell expression of genes and proteins related to the neural development. MAP2, a feature of mature neurons, is frequently used as a marker for neural differentiation, while βIII‐tubulin is acknowledged as one of the early indicators of neuronal differentiation [43, 44]. Additionally, cell nuclei were stained with DAPI to facilitate identification. Immunofluorescence imaging revealed that NPCs cultured on non‐poled + IONPs films (G4) under AMF exposure demonstrated enhanced expression of βIII‐tubulin and MAP2 (Figure 5a–c). Notably, while βIII‐tubulin expression was markedly elevated, MAP2 expression did not show a significant increase in poled films, indicating that the modest thermoelectric effect generated by the poled P(VDF‐TrFE) films (G3) offers limited enhancement in cellular differentiation. Furthermore, the most pronounced differentiation was observed in the group G5 utilizing poled films embedded with IONPs. Composite immunofluorescence imaging confirmed the presence of all three markers, underscoring extensive neuronal network formation under these conditions. Importantly, G4 is not a purely thermal condition: although the film is non‐poled, heating of PVDF domains generates local pyroelectric charges without macroscopic alignment that can still stimulate cells. To isolate the thermal component, we also investigated an α‐type non‐poled P(VDF‐HFP)+IONPs film (G6) that strictly behaves as a thermal emitter. We verified robust heating and precise thermal control with this material. The temperature–time traces showed stable AMF heating, and a tightly regulated 37–41°C temperature window was reproducibly achieved (Figure S11a). Flow cytometry after 7 d at 37–41°C and 20 mT demonstrated that NPCs predominantly adopted a neuronal fate in the poled composite group G5 (poled P(VDF‐TrFE)+IONPs), with approximately 85% neurons and 10% astrocytes, the highest neuronal yield among all groups (Figure S11c–e). Under the same ΔT of 4°C, G4 reached 67% neurons, whereas the strictly thermal G6 reached 52% neurons, indicating that local pyroelectric signals in G4 enhance the effect of heat alone. Reducing field strength to 10 mT or narrowing the thermal window to 37–39°C (Figure S11b) uniformly diminished neuronal differentiation across groups, underscoring a cooperative contribution of thermal dose and magnetopyroelectrical signaling to neuronal specification.

Western blot quantification of MAP2 and βIII‐tubulin corroborated the imaging results (Figure 5d–f). Consistent with the above, βIII‐tubulin was upregulated in G4 under AMF, reflecting the combined influence of heat and locally generated pyroelectric charges in the non‐poled composite. In contrast, the poled film without IONPs (G3; poled P(VDF‐TrFE) only) did not show a significant increase in MAP2, consistent with its limited pyrostimulation in the absence of magnetothermal input. By comparison, the poled composite with IONPs (G5) exhibited pronounced expression of both markers, aligning with the extensive neuronal network formation visualized in merged fluorescence images. Together with the thermal‐only benchmark (G6), these data indicate that pyrostimulation alone promotes differentiation to a certain extent, whereas the additional, aligned electrical stimulation provided by the poled magnetopyroelectric composite synergizes with heat to substantially enhance NPC differentiation.

To assess functional maturation, we applied a prolonged AMF regimen (37–41°C at 20 mT for 10 d) and performed whole‐cell patch‐clamp recordings of poled P(VDF‐TrFE) + IONPs treated NPCs. As shown in Figure S12, approximately 31% of NPC‐derived neurons exhibited spontaneous action potentials, indicating activation of intrinsic excitability. An additional 48% displayed membrane potential oscillations that did not reach full spike threshold but nonetheless reflected increased activity compared with baseline, in which 98% of cells were quiescent. These electrophysiological data substantiate that extended AMF stimulation promotes not only neuronal lineage commitment but also the acquisition of functional properties.

**FIGURE 6:**
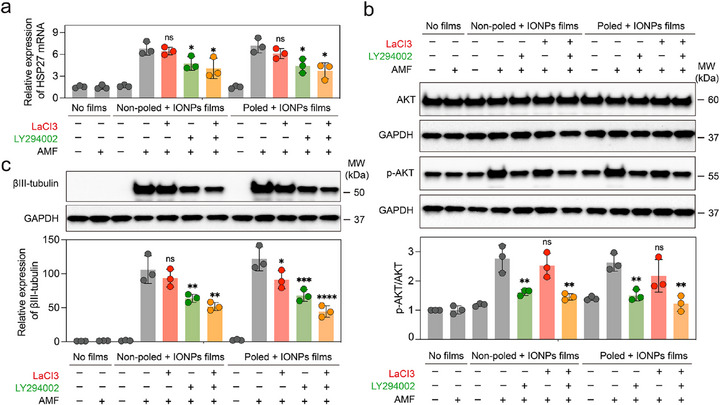
Molecular mechanism of NPC differentiation. (a) Total RNA was extracted from each culture, converted to cDNA, and subjected to real‐time PCR using specific primers targeting HSP27 (n = 3). (b) Western blot assays were conducted, accompanied by quantitative analyses, to assess the expression levels of specific markers (AKT and p‐AKT) following various treatments (n = 3). (c) Similarly, western blot assays and quantitative analyses were performed to evaluate the expression levels of the neural marker βIII‐tubulin after differing treatments (n = 3). Statistical significance was calculated via one‐way ANOVA with a Tukey post‐hoc test (Figure 6a–c).^*^ p < 0.05, ^**^ p < 0.01, ^***^p < 0.001, ^****^ p < 0.0001 versus control.

Then, to investigate the molecular mechanism of cell differentiation, we focused on the calcium signaling and the PI3K‐AKT pathway, both are well‐documented to facilitate cell differentiation [42, 45, 46, 47, 48]. To dissect the pathways involved further, we introduced pathway inhibitors, LaCl_3_ [49], a calcium channel blocker, and LY294002, a PI3K inhibitor [50], to obstruct these signaling routes, together with the AKT activator SC79 [51] and the Ca^2+^ ionophore ionomycin for rescue [52]. Our experiments utilized both non‐poled and poled films embedded with IONPs, which both exhibited clear cell differentiation. Initially, we assessed the involvement of heat shock proteins (HSP)‐key molecular chaperones for cell survival and development. Specifically, the heat‐inducible HSP27 was noted for its potential interaction with the PI3K/Akt signaling pathway [42, 53]. In our experiments with non‐poled and poled IONPs groups under AMF conditions, we discerned distinct effects of pathway inhibition on NPCs differentiation. Quantitative gene expression analysis via real‐time polymerase chain reaction (PCR) enabled precise monitoring of gene activity, revealing that both poled and non‐poled films significantly enhance HSP27 gene expression under thermal stimulation (Figure 6a). As shown in Figure 6b, c, in the non‐poled group, the addition of LaCl_3_ alone did not alter the expression levels of HSP27, p‐AKT (phosphorylated AKT)/AKT, or βIII‐tubulin, indicating that Ca^2+^ channel blockade has minimal impact in the absence of macroscopic electrical alignment. In contrast, LY294002 significantly reduced all three readouts, with βIII‐tubulin decreased by about 40%, and co‐administration of LaCl_3_ produced no further suppression (Figure 6b,c). In the poled composite group, which experiences both heat and aligned pyroelectric currents, LaCl_3_ alone reduced βIII‐tubulin by about 25%, while LY294002 alone significantly reduced HSP27, p‐AKT/AKT, and βIII‐tubulin expression, among them, with βIII‐tubulin decreased by 44%; the combination of LY294002 and LaCl_3_ produced a larger decrease of 64%, indicating synergistic interference with differentiation when both axes are blocked. Crucially, rescue experiments reverse these inhibitory effects and thereby supporting a mechanistic role for the pathway. SC79 restored AKT signaling after LY294002 and recovered neuronal yield toward the poled composite control in the same AMF regimen. Ionomycin similarly rescued the effect of LaCl_3_ by re‐elevating cytosolic Ca^2^^+^ and reinstating βIII‐tubulin expression and neuronal percentage. These mirrored rescue responses are reported in Figure S13 and demonstrate that each pathway is not only permissive but mechanistically required during magnetopyroelectric stimulation. Together, because LaCl_3_ leaves the non‐poled group unchanged, the data indicate that Ca^2^^+^ entry is triggered by the polarization‐dependent electrical cue, and that AKT is needed in both thermal‐only and thermal‐plus‐electrical regimes.

## 3 Conclusions

In this work, we have investigated neural progenitor differentiation strategy driven by heat‐mediated magnetoelectric effect achieved through the synergistic integration of pyroelectricity from P(VDF‐TrFE) and magnetic particle heating by IONPs. This combination has enabled the fabrication of films that exhibit magnetoelectric coupling when exposed to a low‐magnitude, high‐frequency magnetic field. The viability assays conducted with human iPSC‐derived NPCs on these films confirmed their high biocompatibility after 48 h of exposure. Additionally, our studies show that magnetopyroelectric stimulation distinctly promotes the differentiation of NPCs into neurons, and benefits from the synergistic effect of combined heat and electrical stimulation. While pyrostimulation alone does facilitate some degree of differentiation, the concurrent application of electrical stimulation via poled magnetopyroelectric films amplifies this effect, demonstrating a clear synergistic enhancement of NPC differentiation, as evidenced by changes in morphology, flow cytometry, electrophysiological analysis, Western blot analysis, and immunostaining. Pharmacological and molecular data support a mechanism in which calcium signaling and AKT activation mediate the pro‐differentiation response to magnetopyroelectric stimulation. Future studies should further explore electrophysiology to capture acute single channel responses to individual magnetopyroelectric pulses. From a translational standpoint, tissue‐scale field delivery is achieved with kHz frequencies and modest amplitudes that penetrate centimeters with limited attenuation, while coil geometry and duty cycle limit off‐target heating. Safety during prolonged AMF exposure is managed by closed‐loop thermal control and operation within international exposure guidelines. Importantly, PVDF‐based implants are already used clinically, including in hernia‐repair meshes, surgical sutures, and glaucoma drainage devices, owing to their biocompatibility, chemical inertness, and mechanical robustness. Remaining requirements for future translation include long‐term stability and MRI compatibility. These findings underline the potential of the developed composite materials in applications such as tissue engineering, regenerative medicine, and disease treatment, where localized and controlled action is crucial. Moreover, due to the polymeric nature of P(VDF‐TrFE), the composites could be shaped into 3D scaffolds and nanoparticles for further applications in tissue engineering, potentially via methods like electrospinning or electrospraying. As research in this field advances, integrating these materials into clinically relevant applications promises to enhance therapeutic efficacy, providing safer, more efficient, and highly targeted therapeutic options.

## Author Contributions

H.Y. and J.W. contributed equally to this work. The study was designed by H.Y., X.C., and S.P. Magnetopyroelectric materials and setup were fabricated and characterized by J.W. All biology tests were performed by H.Y. Results analysis was undertaken by H.Y. and J.W., with guidance from S.P. The manuscript was written by H.Y. and S.P., and all authors contributed to editing it.

## Conflicts of Interest

The authors declare no conflicts of interest.

## Supporting information




**Supporting File**: advs73911‐sup‐0001‐SuppMat.pdf.

## Data Availability

The data that support the findings of this study are available from the corresponding author upon reasonable request.
