# Supplementary material for: Biocompatible Magnetopyroelectric Composite Films for Cell Stimulation
Source: Adv Sci (Weinh). 2026 Feb 4;13(18):e20491. doi: 10.1002/advs.202520491 (PMC13042573; doi:10.1002/advs.202520491)
Supplement: Supplementary file 1 — Supporting File: advs73911‐sup‐0001‐SuppMat.pdf. [file ADVS-13-e20491-s001.pdf]

Supporting Information

Biocompatible Magnetopyroelectric Composite Films for Cell Stimulation

Hao Ye[1], Joaquin Llacer-Wintle[1], Semih Sevim[1], Elric Zhang[1], Denis von Arx[1], Lukas Hertle[1], Martina Accursi[1], Minsoo Kim[1], Josep Puigmartí-Luis[2,3], Bradley J. Nelson[1], Xiang-Zhong Chen[4,5], Salvador Pané[1*]*

[1] Multi-Scale Robotics Lab (MSRL), Institute of Robotics & Intelligent Systems (IRIS), ETH Zurich, Zurich 8092, Switzerland.

[2] Departament de Ciència dels Materials i Química Física Institut de Química Teòrica i Computacional, University of Barcelona, Barcelona 08028, Spain

[3] Institució Catalana de Recerca i Estudis Avançats (ICREA), Pg. Lluís Companys 23, Barcelona 08010, Spain

[4] International Institute for Intelligent Nanorobots and Nanosystems, College of Intelligent Robotics and Advanced Manufacturing, State Key Laboratory of Photovoltaic Science and Technology, Shanghai Frontiers Science Research Base of Intelligent Optoelectronics and Perception, and Institute of Optoelectronics, Fudan University, Shanghai, 200438, China

[5] Zhejiang Key Laboratory of Extreme Environment Functional Materials, Yiwu Research Institute of Fudan University, Yiwu, 322000, China

EXPERIMENTAL SECTION

Synthesis and characterization of magnetic nanoparticles. Magnetic iron oxide nanoparticles (IONPs) were prepared via thermal decomposition of iron oleate, employing an in-situ temperature marker that flags an endothermic event followed by a rise of temperature coinciding with a translucent-to-opaque color change (nucleation), allowing standardized reaction timing across batches following previously established protocols[1-3]. Initially, iron oleate was produced by reacting $FeCl_3 \cdot 6H_2O$ (2.162 g, 8 mmol, Sigma-Aldrich CAS: 10025-77-1) with sodium oleate (7.309 g, 24 mmol, abcr CAS: 143-19-1). This reaction occurred in a solvent mixture comprising deionized water (12 mL), ethanol (16 mL, Thommen-Furler AG CAS: 64-17-5), and hexane (28 mL, Sigma-Aldrich CAS: 110-54-3), and was refluxed at 70 °C for 4 hours with continuous stirring. Post-synthesis, the iron oleate complex was isolated, extensively purified with water, and aged for six days under atmospheric conditions in an open glass container with stirring at 700 rpm. For nanoparticle formation, aged iron oleate (7.43 mmol) was combined with sodium oleate, oleic acid (Sigma-Aldrich CAS: 112-80-1), and eicosane (Sigma-Aldrich CAS: 112-95-8) in a 100 mL three-neck round-bottom flask. The components were maintained at a molar ratio of 1:0.5:0.825:17.7 respectively, thoroughly mixed at 50 °C and 700 rpm overnight. Subsequently, the system was purged with N_2 and the temperature was incrementally raised to 345 °C at a rate of 3 °C/min, maintained at this temperature for 30 minutes, and then allowed to cool to ambient temperature. To enhance the magnetic properties, the nanoparticles were annealed at 150 °C for 2 hours in air, with continuous stirring. The final purification involved multiple washes with a 10:1 acetone:hexane mixture and a 3:1 methanol:chloroform mixture to remove residual organics. For phase transfer to more polar solvents, the surface ligands of the nanoparticles were exchanged from oleic acid to 3,4-dihydroxyhydrocinnamic acid (DHCA, Sigma-Aldrich CAS: 1078-61-1) following a proven ligand exchange protocol[4]. Hydrophobic nanoparticles (100 mg) were dispersed in THF (5 mL, Sigma-Aldrich CAS: 109-99-9), and DHCA was dissolved separately in THF (30 mL). Both solutions were deoxygenated through successive vacuum and N_2 cycles before the nanoparticle dispersion was added dropwise to the preheated DHCA solution. The mixture was stirred at 50 °C for 3-5 hours, allowed to cool gradually, and the nanoparticles were precipitated using NaOH (0.5-1 mL, 0.5 M, Sigma-Aldrich CAS: 1310-73-2). After several washes with water to neutral pH, the nanoparticles were stored in water, with a recommended storage time of no longer than two weeks.

Transmission electron microscopy (TEM) analyses were conducted using a FEI Talos F200X (Chem S/TEM) operating at 200 kV. This system is equipped with an X-FEG emitter and a CETA camera, which features a 16 M pixel CMOS sensor. For the analysis, the synthesized nanoparticles were first diluted in hexane, then a droplet of this suspension was deposited onto a carbon-coated 400 mesh TEM grid. Subsequently, the grid was allowed to dry in ambient air conditions.

Magnetic properties were assessed utilizing a vibrating sample magnetometer (VSM) provided by MicroSense. For sample preparation, the powder comprising the magnetic particles, previously utilized for X-ray diffraction analysis, was accurately weighed. This powder was subsequently incorporated into melted eicosane to form a solid pellet, wherein the magnetic particles were embedded and stabilized at room temperature.

In a controlled experiment to measure specific loss power (SLP), a homogeneous dispersion of IONPs in aqueous medium, at a concentration of 5 mg mL^{-1}, was subjected to an alternating magnetic field (AMF) with parameters set at a frequency of 500 kHz and a magnetic field strength of 20 mT ($\mu_0 H$). Temperature variations within the sample were meticulously recorded using an optic temperature sensor (Osensa PRB-G40-01M-STM-MRI), positioned centrally in relation to the field. The calculation of SLP was derived from the equation $SLP = \frac{C}{m}\frac{dT}{dt}$, where C represents the specific heat capacity of the dispersion per unit volume, and m denotes the concentration of iron oxide. The assessment of temperature change was intentionally limited to the interval from 4 s to 10 s post-exposure initiation, to circumvent the effects of the initial non-linear thermal response. For the purposes of this evaluation, it was presumed that the system maintained adiabatic conditions throughout the specified time frame, with the specific heat capacity approximated to that of water ($C \approx C_{water} = 4.184$ J K^{-1} mL^{-1}.[5]).

The pyroelectric properties of the composite films were evaluated utilizing a bespoke experimental arrangement founded on the Sharp-Garn technique. The analysis involved films measuring 13 x 38 mm, with an electrode layer of 8 x 30 mm applied to each side using conductive silver paint. During the measurements, each sample was affixed atop a thermoelectric module, with its electrodes connected to a Keithley 6517A electrometer. Temperature control was achieved through a PID feedback mechanism that modulated the sample's temperature in a sinusoidal pattern characterized by an amplitude of 1°C, a frequency of 0.01 Hz, and a temperature ramp of 7.2°C per hour to maintain thermal stability. Concurrently, temperature and electrical current data were captured and analyzed using a

Raspberry Pi system. This setup enabled the calculation of the pyroelectric coefficient as a function of temperature, as delineated in Equation below.

$$p(T) = \frac{I_{AMP} \times sin\emptyset}{A \times \omega \times T_{AMP}} \text{ (Eq. 1)}$$

In this context, I_{AMP} represents the current amplitude, $\emptyset$, A and T_{AMP} denotes the phase offset between the current and temperature, and electrode area.

Fabrication and characterization of MPE films. Pyroelectric films were synthesized through a series of annealing and poling processes applied to drop-casted poly(vinylidene fluoride-co-trifluoroethylene), P(VDF-TrFE). Initially, calculations based on the density of P(VDF-TrFE) (1.9 g cm^{-3}) and the dimensions of the glass substrate (26 x 76 mm) determined that 0.19 g of P(VDF-TrFE) was required to achieve film thicknesses approximately 50 μm. P(VDF-TrFE) (70/30 ratio) provided by Piezotech FC30, was dissolved in dimethylformamide (DMF) (Sigma-Aldrich, CAS: 68-12-2) at a 10 wt% concentration. The mixture of IONPs and P(VDF-TrFE) in DMF was extended over a glass substrate using a doctor blade, to obtain larger samples with better thickness control. Setting the height of the blade to 1 mm resulted in films of approximately 50 μm in thickness and dried at 60 °C for over three hours in a ventilated oven. Subsequent to drying, the films underwent an overnight annealing at 120 °C. Post-annealing, the films were carefully detached from the substrate and sectioned into pieces measuring 13 x 38 mm. A conductive electrode (8 x 30 mm) was then applied centrally on each film using silver paint (PELCO Colloidal Silver 16034). Poling was conducted at room temperature for 5 mins under a corona electric field of 13 kV, maintaining a needle-film distance of 2 cm. During this process, the electrode was grounded, and the opposing film surface faced the charged needle. To preserve the poling effect, the electric field was sustained while the films were cooled to ambient temperature. For the integration of magnetic nanoparticles, identical procedures were followed; however, the P(VDF-TrFE) powder was initially blended with a DMF dispersion containing pre-weighed magnetic nanoparticles. Prior to addition, the IONPs, originally stored in aqueous media, were meticulously cleaned with DMF. This mixture was gently homogenized, carefully avoiding magnetic stirring to prevent nanoparticle aggregation. Additionally, unpoled α-phase poly(vinylidene fluoride-co-hexafluoropropylene), P(VDF-HFP) films containing 10 wt% IONPs were prepared by solution casting from a ketone solvent. P(VDF-HFP) (Sigma-Aldrich, 427179) was dissolved in acetone to 10 wt% and

mixed with IONPs under sonication. The mixture was doctor-bladed onto glass with a 1-mm gap at room temperature, allowed to evaporate in a fume hood, dried at 40 °C, and mildly annealed at 120 °C for 45 min. No mechanical stretching or electrical poling was applied to preserve the α-phase. Phase assignment was verified by XRD.

The magnetopyroelectric properties were evaluated using a bespoke experimental setup. Sample preparation mirrored the protocol utilized for pyroelectric coefficient assessments, wherein each specimen was secured within acrylic holders designed to expose only the electrode regions to ambient air currents. Electrical contacts were established using slender segments of copper foil extending from each holder, which were subsequently linked to a Keithley 6517A electrometer. Positioned centrally within an induction coil supplied by NanoTherics (magneTherm), the sample was subjected to alternating magnetic field pulses ($f = 500$ kHz, $\mu_0 H = 20$ mT, $f_{pulse} = 0.01$-2 Hz), facilitating periodic heating and subsequent cooling phases. The resultant pyroelectric current or voltage was recorded via the electrometer, while an infrared camera (Fluke Ti Infrared Camera) continuously monitored the temperature dynamics. Additionally, resistors, when incorporated in the experiments, were positioned at a considerable distance from the induction coil to prevent interference. Copper foils and connecting wires were meticulously aligned parallel to the magnetic field direction to minimize electrical noise and avoid induction heating effects.

Cell culture. Human induced pluripotent stem cell (iPSC)-derived neural progenitor cell (NPC) culture necessitates the use of Matrigel-coated culture apparatus. The protocol begins with the thawing of Matrigel at low temperatures, followed by a 1:50 dilution in cold DMEM medium. This mixture is then applied to 6-well plates and culture flasks, which undergo overnight incubation at 2-8°C. Prior to cell seeding, the coating solution is aspirated and replaced with ENStem-A Neural Expansion Medium (SCM004a, Merck). Cryopreserved cells (SCC035, Merck) are rapidly thawed at 37°C, then transferred into a sterile 15 mL conical tube where they are gently mixed with pre-warmed medium to mitigate osmotic shock. Subsequent to centrifugation and removal of the supernatant, cells are resuspended and viability is assessed before plating onto the prepared Matrigel-coated surfaces. Cultures are maintained in a 37°C, 5% CO_2 humidified incubator, with media refreshed bi-daily. Upon achieving confluence, NPCs are dissociated using Accutase (Merck, SCR005), counted, and replated into similarly prepared T75 flasks to promote sustained proliferation and maintenance of neural progenitor cells.

Heat-mediated magnetoelectric cell stimulation and setup. The stimulation of magnetopyroelectric cells was carried out using a custom-built setup. The experimental setup involved 26 x 26 mm square films, arranged between a glass slide and a multiwell silicon chamber (Ibidi 12-well chamber, removable). This setup divided the composite film's surface into four sealed com- partments: three were allocated for experimental replicates, and one for monitoring the temperature of the liquid medium with an optical temperature sensor (Osensa PRB-G40-01M-STM-MRI). Each film sample, mounted on the glass slide, was centrally positioned inside an induction coil (manufactured by magneTherm, NanoTherics). Importantly, this induction coil was located within an incubator (New Brunswick Galaxy 170 S) set at a constant temperature of 37°C and an atmosphere of 5% CO2 to simulate physiological conditions and ensure cell viability throughout the experiment. Cell stimulation was achieved by applying periodic pulses (Tpulse = 708 s, AMFON duty cycle = 16.1%) of alternating mag- netic field (f = 500 kHz, $\mu_0 H$ = 20 mT). The selected pulse period and duty cycle were carefully chosen to limit temperature fluctuations of the cell medium between 37°C and 41°C. Temperature profiles for each sample type were recorded, showing the effective management of temperature variations. The procedure for cell stimulation extended up to 10 d, with each day including two 3-hour stimulation sessions, one in the morning and another in the afternoon.

Cell viability. Cell viability was assessed using NPCs. The experimental groups included Non-poled P(VDF-TrFE) films, Poled P(VDF-TrFE) films, Non-poled P(VDF-TrFE) + IONPs films, and Poled P(VDF-TrFE) + IONPs films. Each sample underwent sterilization via ultraviolet radiation for three hours and was then immersed in a 10% solution of penicillin-streptomycin and Amphotericin B, obtained from Sigma Aldrich. This was followed by thorough rinsing in phosphate-buffered saline. The sterilized films were placed into individual wells of a 12-well plate (ibidi, 81201) to support cell culture. NPCs were seeded at a density of 1×10^4 cells per well for subsequent MTT (3-(4, 5-dimethylthiazol-2-yl)-2,5-diphenyltetrazolium bromide) assay. Twenty-four hours post-seeding, the films served as substrates to which the cells adhered. Each group was then exposed to cycles of an applied AMF and non-applied AMF, twice daily for two days. To assess long-term biocompatibility, we stimulated and evaluated the NPCs over a 10 d period under various conditions. Cell viability was evaluated using the MTT assay with a tetrazolium salt

concentration of 3 mg/mL. Absorbance was measured at 570 nm with a reference wavelength of 630 nm using a Varioskan Flash multimode microreader. The mean absorbance from triplicate wells was used to calculate cell viability:

$$Cell\ viabiliy\ of\ NPC\ \%= \left(\frac{OD_{sample}\text{-}OD_{blank}}{OD_{control}\text{-}OD_{blank}}\right) \times 100$$

where OD_sample represents the optical density of treated cells, OD_blank is the baseline optical density from the cell culture medium without cells, and OD_control is from NPC cells cultured without film treatment and not exposed to AMF.

In addition to MTT assays, biocompatibility was further evaluated through a live/dead assay. Cells were incubated with a live/dead staining solution (Invitrogen, Catalogue Number: R37601) for 15 minutes at room temperature. Post-incubation, samples were imaged with a LSM 880 Airyscan confocal microscope (Zeiss), and images were quantitatively analyzed using ImageJ software. This dual approach ensured a comprehensive assessment of the biocompatibility and cytotoxic effects of the composite films under study.

Magnetopyroelectric stimulation of NPCs. To assess the efficacy of stimulation on NPCs, immunofluorescent staining was conducted following exposure to AMF. Each specimen was subjected to AMF stimulation bi-daily for a duration of two days. Subsequent to stimulation, the cells were fixed with 4% paraformaldehyde (PFA) for 10 minutes at room temperature. This was followed by cell permeabilization using 0.5% Triton X-100 for 30 minutes at room temperature. To mitigate non-specific binding, blocking was performed with 2% bovine serum albumin (BSA) for 30 minutes. Primary antibodies targeting βIII-tubulin (Sigma-Aldrich, T2200) and microtubule-associated protein 2 (MAP2) (Proteintech, 67015-1) were applied, and the samples were incubated overnight at 4 °C. This was followed by incubation with secondary antibodies: FITC-conjugated goat anti-rabbit IgG (Sigma-Aldrich, AP132F) and TRITC-conjugated goat anti-mouse IgG (Sigma-Aldrich, AP503R) for one hour. After several washes and application of nuclear stains, the samples were visualized using a Zeiss LSM 880 Airyscan confocal laser scanning microscope. Image quantification was performed using ImageJ software. The differentiation percentage of NPCs was quantified following stimulation for 2 h twice daily over 2 or 7 d. Post-stimulation, cells were stained with Alexa Fluor® 488-conjugated antibodies against βIII-tubulin (BioLegend, Cat. No. 801203) or GFAP (BD Biosciences, Cat. No. 561449) and analyzed by flow cytometry (LSRFortessa, BD Biosciences). For electrophysiological measurements, NPCs were stimulated over 7 or 10

d. Electrophysiological activity was then recorded using whole-cell patch-clamp techniques, utilizing an Axopatch 200 amplifier, a Digidata 1322A interface, and Clampex 10.2 software (Molecular Devices).

Western blot assay. Protein extraction from cultured cells was conducted using Thermo Scientific™ RIPA Lysis and Extraction Buffer (Catalog number: 89900), enhanced with PMSF Protease Inhibitor (Catalog number: 36978) to optimize protein yield and inhibit proteolytic degradation. Protein concentrations were determined utilizing the Thermo Scientific™ Pierce™ BCA Protein Assay Kit (Catalog number: 23227). Proteins were then separated by SDS-PAGE, employing Tris-Glycine-SDS Buffer (Sigma, T7777) adjusted to the appropriate working concentration. Post-electrophoresis, the proteins were translocated to polyvinylidene fluoride (PVDF) membranes using Thermo Scientific™ Pierce™ 10X Western Blot Transfer Buffer, Methanol-free (Catalog number: 35040). To minimize nonspecific interactions, membranes were blocked with Thermo Scientific™ Pierce™ Clear Milk Blocking Buffer (10X), diluted to a 1X working solution (Catalog number: 37587), for one hour at ambient temperature. Membranes were then incubated overnight at 4°C with primary antibodies against bIII-tubulin (Sigma, T2200), MAP2 (Abcam, ab32454), AKT (Proteintech, 10176-2-AP), and Phospho-AKT (Proteintech, 80455-1-RR) following the recommended dilutions provided by the manufacturers. Subsequent to primary antibody exposure, the membranes underwent washing and were exposed to horseradish peroxidase (HRP)-linked secondary antibodies (Sigma, AP307P) for one hour at room temperature. Detection of protein bands was achieved using Clarity™ Western ECL Substrate (Bio-Rad, 200 ml #1705060) and visualized on an Bio-Rad imaging system (ChemiDoc XRS+), utilizing ImageJ software for digital rendering and quantitative assessment.

Statistical analysis. All results were indicated as mean ± standard error of the mean (s.e.m.). One-way ANOVA (Tukey post-hoc tests) were applied for multiple comparisons and the Kruskal–Wallis non-parametric test with Dunnett's post hoc analysis was used to analysis the non-normally distributed data. All statistical analyses were conducted using the Prism software package (PRISM 9.5.0; GraphPad Software, 2022). $P < 0.05$ indicated statistical significance.

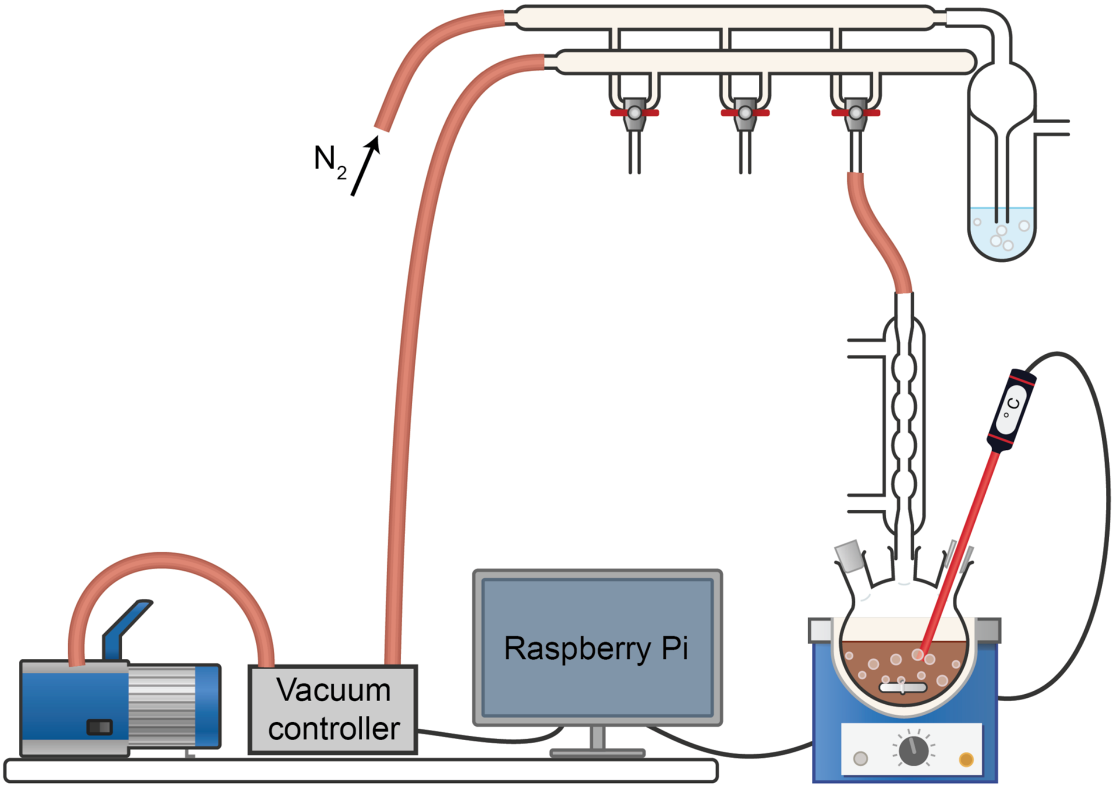

Figure S1. IONP thermal decomposition setup schematic.

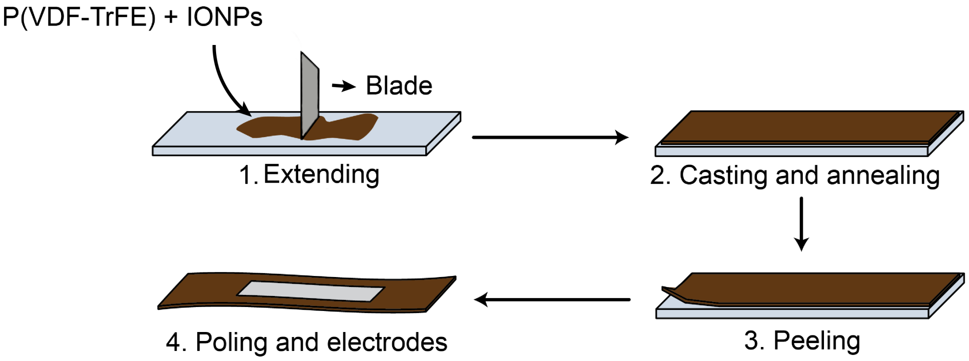

Figure S2. Schematic of the Fabrication Process for MPE Films.

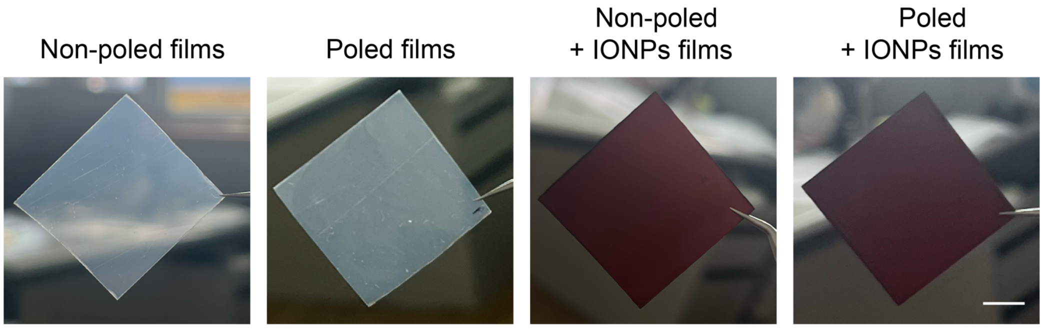

Figure S3. Photographs of the fabricated MPE films. Scale bar = 8 mm.

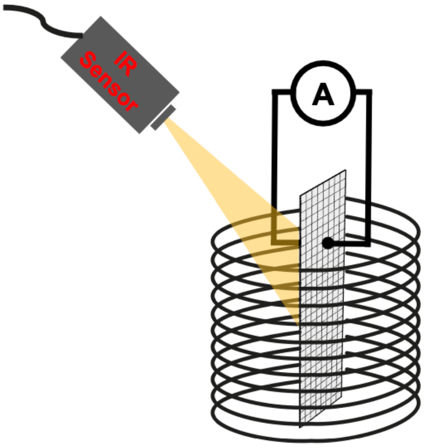

Figure S4. Schematic of the magnetopyroelectric measuring setup scenario.

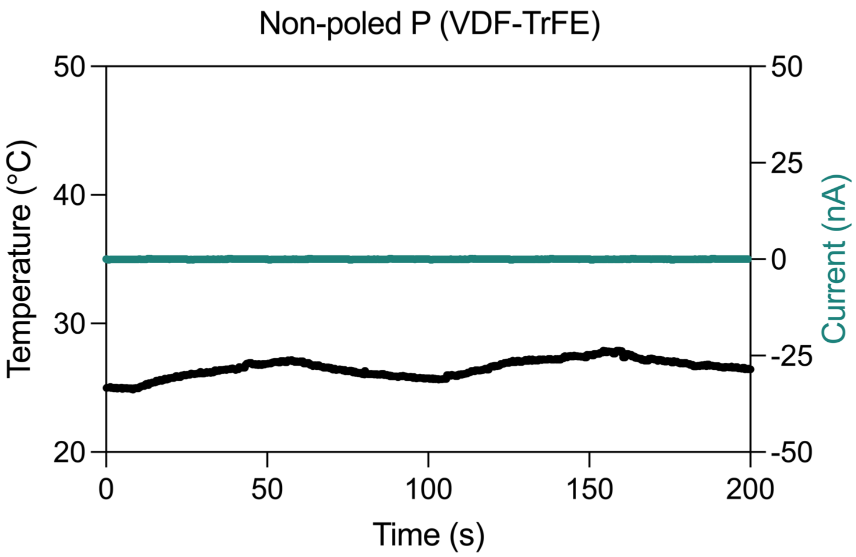

Figure S5. Proof of magnetopyroelectric coupling in the films used for cell stimulation. Upon the application of a pulsed alternating magnetic field (μ_0H = 20 mT, f = 500 kHz, Tpulse = 100 s), the temperature and the current are measured for Non-poled P(VDF-TrFE) film.

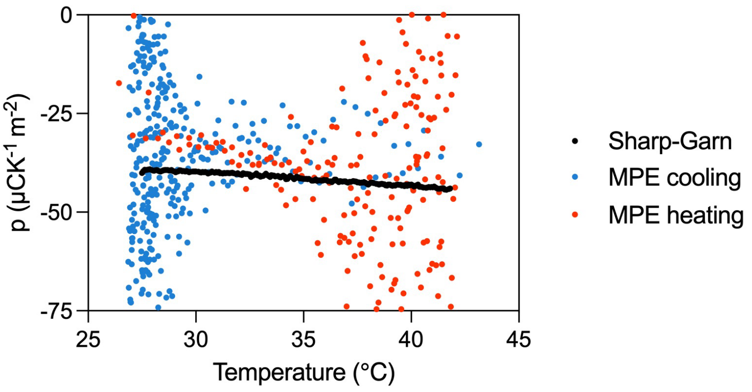

Figure S6. Comparison of pyroelectric coefficients from MPE measurements and from Sharp-Garn measurements. Pyroelectric coefficients are calculated from the heating and cooling steps of the MPE data (Figure 3c,d) according to equation below and compared to pyroelectric coefficient obtained from the Sharp-Garn method.

$$p(T) = \frac{I_P}{A \times dT/dt}$$

where I_P, A and dT/dt denote the pyroelectric current, the area of the measuring electrodes and the temperature change rate

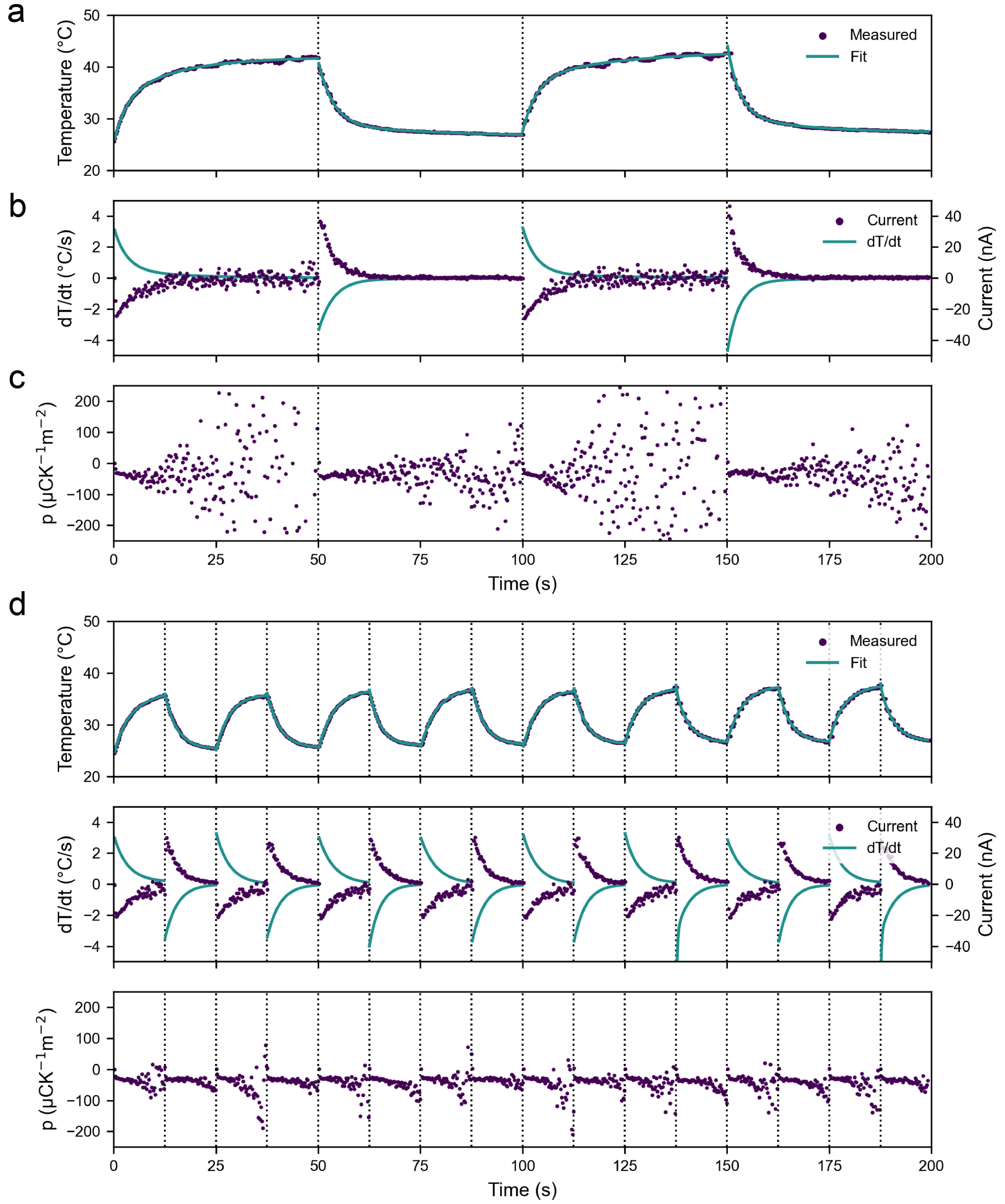

Figure S7. Pyroelectric coefficient calculation from a typical MPE measurement. (a) Two-phase decay fitting of the temperature curves. (b) The time derivative of the fitted temperature and the measured pyroelectric current are used to calculate (c) the pyroelectric coefficient. (d) Observation of pyroelectric activity under another typical conditions.

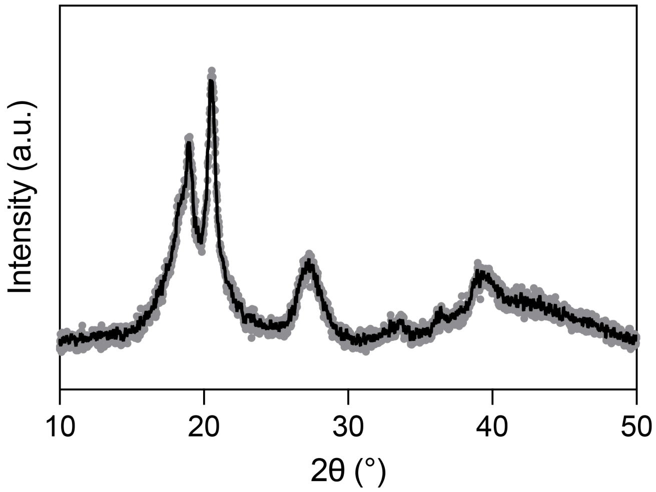

Figure S8. XRD pattern of α-type non-poled P(VDF-HFP) films.

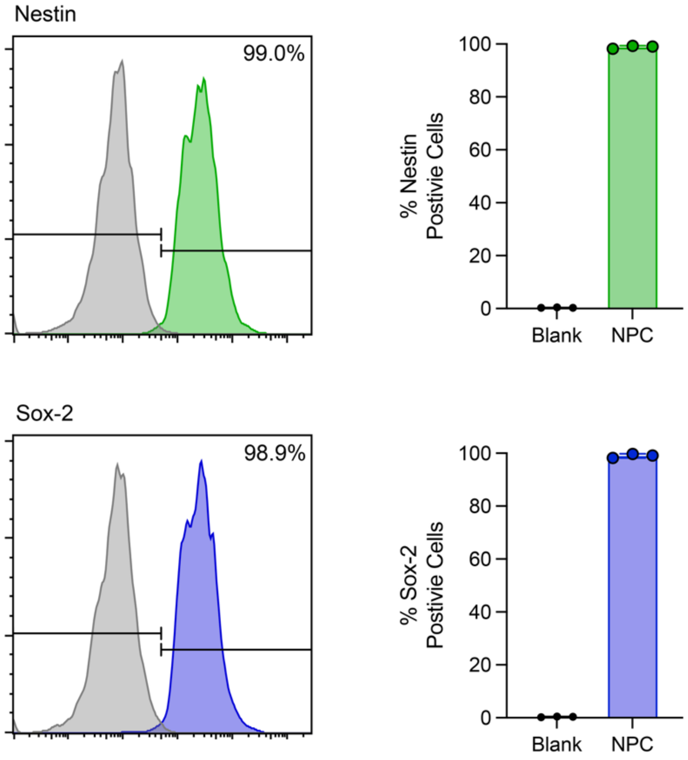

Figure S9. Representative histograms and quantification of the percentage of stem cell marker Nestin, and Sox-2. Means ± s.e.m, n = 3 experiments.

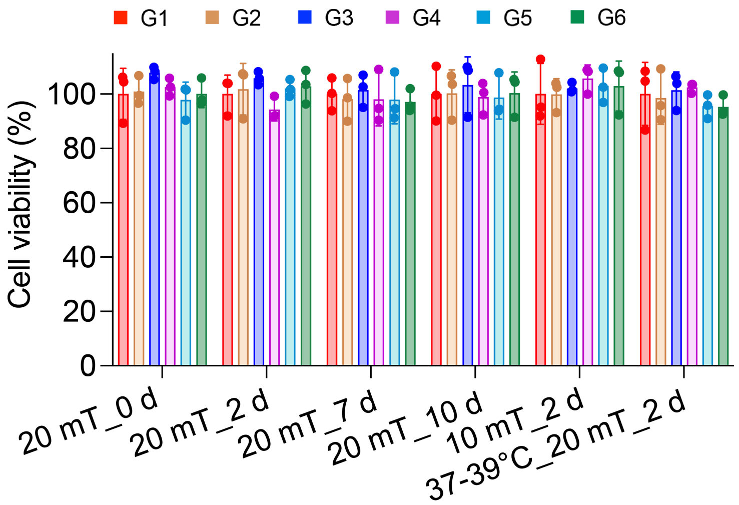

Figure S10. Biocompatibility evaluation of various samples under different conditions over 10 d AMF stimulation by MTT assay. G1: No films; G2: Non-poled P(VDF-TrFE) films; G3: Poled P(VDF-TrFE) films; G4: Non-poled P(VDF-TrFE) + IONPs films; G5: Poled P(VDF-TrFE) + IONPs films; G6: Non-poled P(VDF-HFP) + IONPs films. Means ± s.e.m, n = 3 experiments.

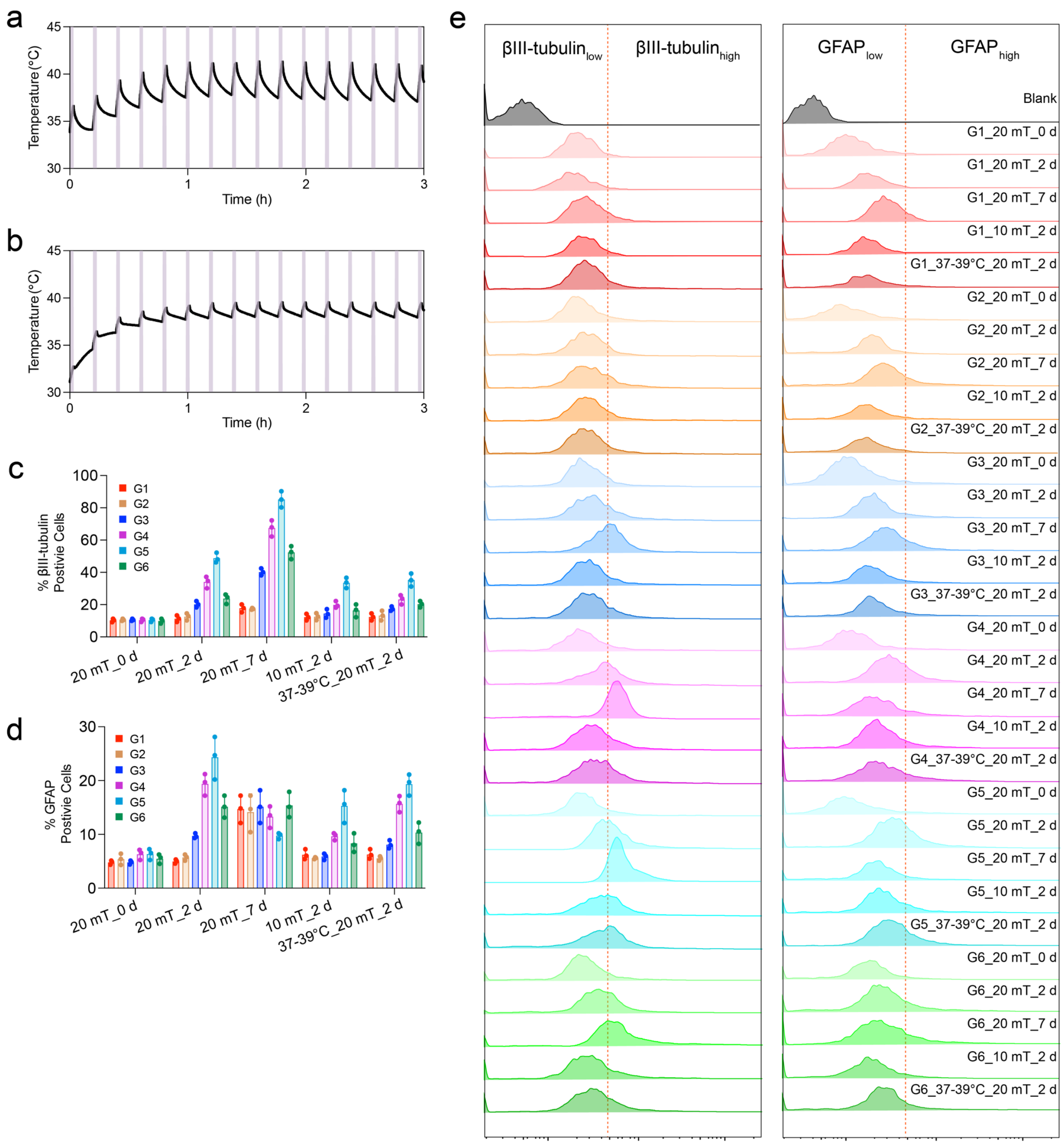

Figure S11. Magnetopyroelectric Stimulation Promotes the Differentiation of NPCs into Neuronal and Astrocytic Lineages. Representative temperature profiles of (a) the non-poled P(VDF-HFP) + IONPs film (G6) and (b) the poled P(VDF-TrFE) + IONPs film during a 37–39 °C stimulation cycle. (c-e) Flow cytometry analysis of NPCs differentiation after 2 and 7 days of stimulation. (c, d) Quantification of the percentage of βIII-tubulin-positive and GFAP-positive cells for each condition. (e) Representative histograms show cell populations stained for the neuronal marker βIII-tubulin and the astrocytic marker GFAP. G1: No films; G2: Non-poled P(VDF-TrFE) films; G3: Poled P(VDF-TrFE) films; G4: Non-poled P(VDF-TrFE) + IONPs films;

G5: Poled P(VDF-TrFE) + IONPs films; G6: Non-poled P(VDF-HFP) + IONPs films. Means ± s.e.m, n = 3 experiments.

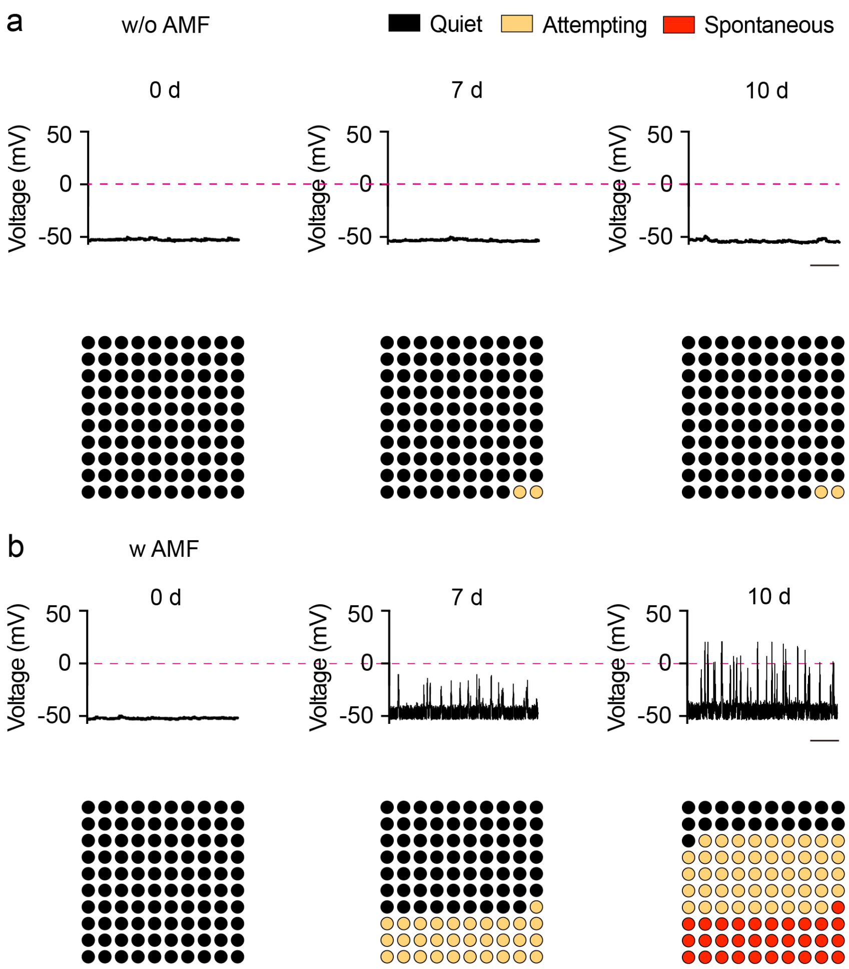

Figure S12. Electrophysiological Recordings of NPCs under Magnetopyroelectric Stimulation. (a) Voltage traces for samples over 0, 7, and 10 days without AMF stimulation. (b) Electrophysiological recordings from poled P(VDF-TrFE) + IONPs films treated samples subjected to AMF stimulation for 7 and 10 days. The emergence of different firing patterns by patch-clamp recording (quiet, attempting (spontaneous action potential-like oscillations below 0 mV), and spontaneous (action potential)) is summarized by the 10×10 grids below each trace, with the state of 100 individual NPCs per condiction. Scale bar = 300 ms

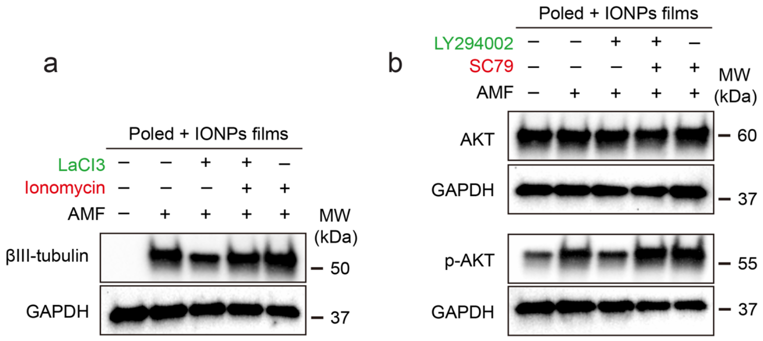

Figure S13. Molecular mechanism of NPC differentiation. Protein expression levels of (a) βIII-tubulin, (b) total AKT, and phosphorylated AKT (p-AKT) were assessed using Western blot assays following various treatment conditions.

References:

[1] E. Wetterskog, M. Agthe, A. Mayence, J. Grins, D. Wang, S. Rana, A. Ahniyaz, G. Salazar-Alvarez, L. Bergstrom, *Sci Technol Adv Mater* **2014**, 15, 055010.

[2] J. Park, K. An, Y. Hwang, J. G. Park, H. J. Noh, J. Y. Kim, J. H. Park, N. M. Hwang, T. Hyeon, *Nat Mater* **2004**, 3, 891.

[3] J. Llacer-Wintle, L. Hertle, S. Ziegler, E. Pellicer, A. G. Roca, J. Nogués, J. Puigmartí-Luis, B. J. Nelson, X. Z. Chen, S. Pané, *Advanced Functional Materials* **2024**.

[4] Y. Liu, T. Chen, C. Wu, L. Qiu, R. Hu, J. Li, S. Cansiz, L. Zhang, C. Cui, G. Zhu, M. You, T. Zhang, W. Tan, *J Am Chem Soc* **2014**, 136, 12552.

[5] S.-Y. Wang, S. Huang, D.-A. Borca-Tasciuc, *IEEE Transactions on Magnetics* **2013**, 49, 255.
